# Reliability and Validity of the 6‐Minute Walk Test in Hypophosphatasia

**DOI:** 10.1002/jbm4.10131

**Published:** 2019-03-01

**Authors:** Dawn Phillips, Ioannis C Tomazos, Scott Moseley, Gil L'Italien, Hugo Gomes da Silva, Sergio Lerma Lara

**Affiliations:** ^1^ Division of Physical Therapy Department of Allied Health Sciences University of North Carolina Chapel Hill NC USA; ^2^ Alexion Pharmaceuticals, Inc. Boston MA USA; ^3^ Centro Superior de Estudios Universitarios (CSEU) La Salle Universidad Autónoma de Madrid Madrid Spain; ^4^ Hospital Infantil Universitario Niño Jesús Madrid Spain

**Keywords:** BONE DISEASES, METABOLIC, QUALITY OF LIFE, AMBULATION, VALIDATION STUDIES, MINIMAL CLINICALLY IMPORTANT DIFFERENCE

## Abstract

This investigation evaluated the reliability and validity of the 6‐Minute Walk Test (6MWT) in patients with pediatric hypophosphatasia (HPP). Children (aged 6 to 12 years; *n* = 11), adolescents (13 to 17 years; *n* = 4), and adults (18 to 65 years; *n* = 9) completed the 6MWT at screening and baseline in two clinical studies of asfotase alfa. Test‐retest reliability of the 6MWT, evaluated with Pearson's correlation coefficients (*r*) for screening versus baseline, was high for children (*r* = 0.95; *p* < 0.0001), adolescents (*r* = 0.81; *p* = 0.125), and adults (*r* = 0.94; *p* = 0.0001). The most conservative minimal clinically important differences, estimated using distribution‐based methods, were 31 m (children and adults) and 43 m (adolescents). In children, the 6MWT correlated significantly with scores on measures of skeletal disease, which included the Radiographic Global Impression of Change scale (*r* = 0.50; *p* < 0.0001) and the Rickets Severity Scale (*r* = −0.78; *p* < 0.0001), such that distance walked increased as the severity of skeletal disease decreased. Significant (*p* < 0.0001) correlations with the 6MWT distance walked were also observed for children with scores on parent‐reported measures of disability (*r* = −0.67), ability to function in activities of daily living (*r* = 0.71 to 0.77), and parent‐reported measures of pain (*r* = −0.39). In adolescents and adults, 6MWT distance walked correlated significantly (*p* < 0.05) with measures of lower extremity function (*r* = 0.83 and 0.60, respectively), total pain severity (*r* = −0.41 and −0.36, respectively), and total pain interference (*r* = −0.41 and −0.49, respectively). Collectively, these data indicate that the 6MWT is a reliable, valid measure of physical functioning in patients with pediatric HPP. © 2018 The Authors. *JBMR Plus* Published by Wiley Periodicals, Inc. on behalf of the American Society for Bone and Mineral Research.

## Introduction

Hypophosphatasia (HPP) is a rare, inherited, systemic metabolic disease characterized by low tissue‐nonspecific alkaline phosphatase (TNSALP) activity.^1^, ^2^ Signs, symptoms, and complications of HPP in children and adults can include impaired skeletal mineralization, HPP rickets, bone deformities, fractures, short stature, pain, muscle weakness, and reduced physical function such as compromises in ambulation.^3^, ^4^, ^5^, ^6^, ^7^ As a result, patients with HPP may have impaired overall functional status, reduced ability to perform activities of daily living, and a lower quality of life.^3^, ^6^ Improvement in physical function is an important goal in the management of HPP.

The 6‐Minute Walk Test (6MWT) is a self‐paced walking test that measures the distance an individual is able to walk on a hard, flat surface for 6 min.^8^ Originally developed for assessment of aerobic activity in patients with respiratory disease,^9^ the 6MWT is now validated in numerous other patient populations,^10^, ^11^, ^12^ including those with musculoskeletal diseases (eg, Duchenne/Becker muscular dystrophy,^13^, ^14^ facioscapulohumeral muscular dystrophy,^15^ spinal muscle atrophy^16^), and has been used as an efficacy measure in clinical studies of patients with muscular^17^, ^18^, ^19^ and metabolic^20^, ^21^, ^22^, ^23^ disorders. The 6MWT has also been used as an outcome measure in clinical studies of patients with other rare diseases, such as Pompe disease,^24^ Hunter syndrome,^25^ and Morquio A syndrome.^26^, ^27^, ^28^ The 6MWT assesses the integrated response of the pulmonary, cardiovascular, and musculoskeletal systems and reflects the functional exercise level required to perform daily life activities.^8^ Normal walking distances for healthy individuals vary with sex, age, weight, and height.^8^, ^29^, ^30^

The reliability, validity, and minimal clinically important difference (MCID; smallest change in distance walked that would be considered clinically meaningful) of the 6MWT have not been established in patients with HPP. The main purpose of this analysis was to establish the MCID of the 6MWT in HPP. In addition, we evaluated the test‐retest reliability of the 6MWT and the concurrent validity of the 6MWT and measures of skeletal disease, ability to perform daily activities, functional disability, and pain.

## Patients and Methods

### Data sources

Data from two clinical studies of asfotase alfa (Strensiq®; Alexion Pharmaceuticals, Inc., Boston, MA, USA), a TNSALP enzyme replacement therapy for the treatment of HPP, were used for these analyses. Study 1 was a Phase 2, open‐label, 6‐month study of asfotase alfa and its 6‐year extension in children aged 6 to 12 years at enrollment (NCT00952484 and NCT01203826).^31^ Study 2 was a Phase 2, open‐label, 6‐month study of asfotase alfa and its 6‐year extension in adolescents and adults aged 13 to 65 years at enrollment (NCT01163149). For both studies, inclusion criteria required serum ALP activity below the age‐adjusted normal range, plasma pyridoxal 5′‐phosphate level at least twice the upper limit of normal, presence of HPP rickets (study 1) or osteopenia/osteomalacia (study 2) on skeletal radiographs, and serum 25‐hydroxy vitamin D level ≥20 ng/mL. This analysis included data from children (study 1: age 6 to 12 years at enrollment), adolescents (study 2: age 13 to 17 years at enrollment), and adults (study 2: age ≥18 years at enrollment) who had first signs and symptoms of HPP before age 18 years (pediatric HPP) and who completed the 6MWT at screening or baseline.

Both studies were conducted in accordance with guidelines from the Declaration of Helsinki on Ethical Principles for Medical Research Involving Human Subjects and International Council for Harmonisation Good Clinical Practice Guidelines. The protocol and informed consent form were reviewed and approved by the institutional review board or research ethics board before study initiation at each investigational site (Biomedical Research Ethics Board, University of Manitoba, Winnipeg, MB, Canada; Human Research Protection Office, Washington University, St. Louis, MO, USA; and Institutional Review Board, Duke University Medical Center, Durham, NC, USA). Patients or their parents/legal guardians provided informed consent before study participation. Qualified academic investigators may request participant‐level, deidentified clinical data and supporting documents (statistical analysis plan and protocol) pertaining to this study. Further details regarding data availability and instructions for requesting information and our data disclosure policy are available on the Alexion.com website (http://alexion.com/research-development).

### Assessments

The 6MWT distances walked at screening and baseline were used to evaluate test‐retest reliability and the MCID. The extent to which the 6MWT correlates with other measures commonly used in HPP was also evaluated. This concurrent validity of the 6MWT was assessed by correlating distance walked with measures of skeletal disease, ability to perform daily activities, and pain in children with HPP and measures of functional disability and pain in adolescents and adults with HPP. These assessments are detailed below and in Table 1.^8^, ^9^, ^10^, ^11^, ^12^, ^13^, ^14^, ^15^, ^16^, ^32^, ^33^, ^34^, ^35^, ^36^, ^37^, ^38^, ^39^

**Table 1:** Summary of Assessments

Assessment	Description
**Walking ability**	
–	
6‐Minute Walk Test (6MWT)^8^	• Originally developed for assessment of aerobic activity in patients with respiratory disease^9^; also validated in numerous other patient populations,^10^, ^11^, ^12^ including those with musculoskeletal diseases (eg, Duchenne/Becker muscular dystrophy,^13^, ^14^ facioscapulohumeral muscular dystrophy,^15^ spinal muscle atrophy^16^). • Patients were instructed to walk a 60‐m lap along the length of a hallway for 6 min, and the total number of meters walked was recorded. • Patients who required assistive devices for ambulation used their usual walking aids (eg, orthotics, walker) during the test.
–	
**Skeletal disease in children with HPP**	
–	
Radiographic Global Impression of Change (RGI‐C) scale^32^	• Validated in newborns, infants, and children with HPP. • Three independent pediatric radiologists rated changes from baseline at each time point. • 7‐point scale (−3 = severe worsening of skeletal features of HPP, 0 = no change, and +3 = complete or near complete healing of skeletal features of HPP).
Rickets Severity Scale (RSS)^33^	• Validated in children with nutritional rickets (mean age: 4.5 years). • One independent rater evaluated radiographs of the wrists and knees from each time point. • 10‐point scale (0 = absence of metaphyseal cupping and fraying to 10 = severe rickets; maximum of 4 points for the wrists and 6 points for the knees).
–	
**Ability to perform activities of daily living and pain in children with HPP**	
–	
Childhood Health Assessment Questionnaire (CHAQ)^34^	• Validated in individuals aged 1–19 years with juvenile rheumatoid arthritis. • Comprises 30 questions in 8 subscales (dressing and grooming, arising, eating, walking, hygiene, reach, grip, and activities) that are scored from 0–3 (0 = without any difficulty, 1 = with some difficulty, 2 = with much difficulty, and 3 = unable to do). ○ The highest score for any item within a subscale determines the score for that subscale. • The CHAQ Disability Index (CHAQ‐DI) is the mean of the scores on each of the 8 subscales, with higher scores indicating greater disability. • The CHAQ Pain Index (CHAQ‐PI) is measured on a visual analog scale ranging from 0 (no pain) to 100 (very severe pain).
Pediatric Outcomes Data Collection Instrument (PODCI)^35^, ^36^	• Validated in pediatric orthopedic patients aged 2–18 years. • A patient‐/parent‐reported instrument used to assess ability to perform activities of daily living (eg, walk 1 block, get out of bed, get on and off the toilet, get on and off a bus). • Four PODCI functional subscales were applied (Upper Extremity and Physical Function, Transfer and Basic Mobility, Sports and Physical Functioning, and Pain/Comfort). ○ These scores are averaged to determine the PODCI Global Functioning score. • Standardized scores for each subscale range from 0 (poorest outcome/worst health) to 100 (best possible outcome/best health). • Normative values were calculated for the 4 subscales and the Global Functioning score and referenced to the general healthy US population (mean ± standard deviation normative score of 50 ± 10 represents a healthy population).
–	
**Functional disability and pain in adolescents and adults with HPP**	
–	
Lower Extremity Functional Scale (LEFS)^37^	• Validated in patients with lower‐extremity musculoskeletal conditions (mean age: 44 years). • Scored 0–80. • Higher scores indicate better lower extremity functioning in daily life activities, including ability to perform in transitional movements (eg, getting out of bath, rolling in bed), locomotion (eg, walking, running on uneven ground, climbing stairs, squatting).
Brief Pain Inventory–Short Form (BPI‐SF)^38^, ^39^	• Validated in a number of conditions, including musculoskeletal conditions. • Includes 4 items that assess pain severity (11‐point scale: 0 = no pain, 10 = worst pain you can imagine) and 7 items that assess pain interference with functioning (11‐point scale: 0 = does not interfere, 10 = completely interferes) in the preceding 24 hours. • A total pain severity score (BPI‐SF‐PS; range: 0–40) is calculated from the 4 items of pain intensity, with each item weighted equally in the final score. • A total pain interference score (BPI‐SF‐PI; range: 0–70) is calculated from the 7 items on pain interference, each contributing the same weight to the final score.

HPP = hypophosphatasia.

#### 6MWT

In study 1 and study 2, patients completed the 6MWT twice before receiving treatment with asfotase alfa, first at the screening visit and again at the baseline visit, which occurred ≥3 weeks after screening in study 1 and ∼7 weeks after screening in study 2. Patients then completed the 6MWT at study visits during the primary treatment period and extension phase. The 6MWT was administered by a physical therapist in accordance with American Thoracic Society guidelines (Table 1).^8^, ^32^, ^33^, ^34^, ^35^, ^36^, ^37^, ^38^, ^39^ Percent predicted values for the 6MWT, defined as the percent of normal predicted distance walked based on sex, age, and height,^29^ were calculated.

#### Measures of skeletal disease in children with HPP

In study 1, changes in HPP rickets severity were evaluated in skeletal radiographs of the bilateral wrists and bilateral knees obtained at baseline and periodically during the primary treatment period and extension phase using the Radiographic Global Impression of Change (RGI‐C) scale validated in patients with HPP^(32)^ and the Rickets Severity Scale (RSS; Table 1).^(33)^

#### Measures of ability to perform activities of daily living and pain in children with HPP

In study 1, parents/caregivers rated children's ability to function while performing activities of daily living at baseline and every study visit using the Childhood Health Assessment Questionnaire (CHAQ)^34^ and the Pediatric Outcomes Data Collection Instrument (PODCI)^35^, ^36^ (Table 1). A parent or legal guardian completed the pediatric version of the PODCI for patients aged <18 years. The adolescent versions of the questionnaire, to be completed by the patient or parent/legal guardian, were also completed for patients aged ≥11 years. Analysis of the results was based on the parent‐reported pediatric assessment.

#### Measures of functional disability and pain in adolescents and adults with HPP

In study 2, patient‐reported functional disability was assessed using the Lower Extremity Functional Scale (LEFS),^37^ and pain was assessed using the Brief Pain Inventory–Short Form (BPI‐SF) (Table 1).^38^, ^39^ The LEFS and the BPI‐SF were administered by a licensed physical therapist at baseline and periodically during the primary treatment period and extension phase.

### Statistical analyses

All analyses were conducted separately for children aged 6 to 12 years, adolescents aged 13 to 17 years, and adults aged ≥18 years. The test‐retest reliability analysis was conducted for the population of patients who completed the 6MWT at both screening and baseline. Pearson's correlation coefficients (*r*) were calculated between 6MWT distances walked at screening versus baseline. Two‐sided *p* values were calculated (exact test null hypothesis: *r* = 0).

An HPP‐specific estimate of the MCID for the 6MWT was calculated using two distribution‐based methods applied to baseline/screening 6MWT data: the standard error of measurement (SEM) method (standard deviation [SD] × √[1–*r*]) and the one‐third SD method (SD × 1/3), following the methods used for patients with Duchenne muscular dystrophy.^14^ The *r* value used in the SEM MCID calculation was calculated from the population of patients who completed the 6MWT at both screening and baseline. The SD values used for both the SEM and the SD methods of calculating the MCID were from the composite baseline population, which included all patients who completed the 6MWT at screening and/or baseline (screening value was used if baseline value was missing).

The concurrent validity analyses were conducted for the population of patients who completed the 6MWT at both screening and baseline. Pearson correlation coefficients were calculated between 6MWT distance walked and scores on measures of skeletal disease (RGI‐C and RSS in children), functional status (CHAQ Disability Index [CHAQ‐DI] and PODCI Global Functioning normative score, PODCI Transfers and Basic Mobility subscale normative score, and Sports and Physical Functioning subscale normative score in children and LEFS in adolescents and adults), and pain (CHAQ Pain Interference [CHAQ‐PI] and PODCI Pain/Comfort subscale normative score in children and BPI‐SF severity and interference totals in adolescents and adults).

## Results

### 6MWT MCID, test‐retest reliability, and concurrent validity

#### Children

Disposition and demographics of the 13 children with HPP enrolled in study 1 are summarized in Table 2. Two children used assistive devices (orthotic shoe inserts, *n* = 2) during the 6MWT at screening and baseline.

**Table 2:** Demographics and Baseline Characteristics

Patients with HPP (age at enrollment)	Completed 6MWT at screening and BL	Did not complete 6MWT at screening and BL	Overall
–			
**Children (5–12 years)**	*n* = 11	*n* = 2^a^	*n* = 13
–			
Age at enrollment (years)			
Mean ± SD	8.9 ± 2.2	8.0 ± 2.9	8.8 ± 2.2
Median (minimum, maximum)	8.6 (6, 12)	8.0 (6, 10)	8.6 (6, 12)
Age at onset of signs, symptoms, and/or complications of HPP (years)			
Mean ± SD	1.0 ± 0.6	0.3 ± 0.1	0.9 ± 0.6
Median (minimum, maximum)	1.0 (0.1, 1.8)	0.3 (0.3, 0.4)	1.0 (0.1, 1.8)
Male, *n* (%)	9 (81.8)	2 (100)	11 (84.6)
Race, *n* (%)			
White	11 (100)	1 (50.0)	12 (92.3)
Other	0	1 (50.0)	1 (7.7)
Ethnicity, *n* (%)			
Hispanic or Latino	1 (9.1)	0	1 (7.7)
Not Hispanic or Latino	10 (90.9)	2 (100)	12 (92.3)
**Adolescents (13–17 years)**	***n* = 4**	***n* = 2** ^b^	***n* = 6**
Age at enrollment (years)			
Mean ± SD	15.8 ± 0.9	13.7 ± 1.0	15.1 ± 1.3
Median (minimum, maximum)	15.8 (15, 17)	13.7 (13, 14)	15.1 (13, 17)
Age at onset of signs, symptoms, and/or complications of HPP (years)			
Mean ± SD	0.3 ± 0.3	0.6 ± 0.6	0.4 ± 0.4
Median (minimum, maximum)	0.3 (0, 0.5)	0.6 (0.2, 1.0)	0.3 (0, 1.0)
Male, *n* (%)	2 (50.0)	2 (100)	4 (66.7)
Race, *n* (%)			
White	4 (100)	1 (50.0)	5 (83.3)
Other	0	1 (50.0)	1 (16.7)
Ethnicity, *n* (%)			
Not Hispanic or Latino	4 (100)	2 (100)	6 (100)
**Adults (≥18 years)**	***n* = 9**	***n* = 1** ^c^	***n* = 10**
Age at enrollment (years)			
Mean ± SD	55.0 ± 3.8	26.5 ± NA	52.2 ± 9.7
Median (minimum, maximum)	55.5 (46, 59)	26.5 (NA)	55.5 (27, 59)
Age at onset of signs, symptoms, and/or complications of HPP (years)			
Mean ± SD	2.2 ± 1.2	2.0 ± NA	2.2 ± 1.1
Median (minimum, maximum)	2.0 (0.1,4.0)	2.0 (NA)	2.0 (0.1,4.0)
Male, *n* (%)	1 (11.1)	1 (100)	2 (20.0)
Race, *n* (%)			
White	9 (100)	1 (100)	10 (100)
Ethnicity, *n* (%)			
Not Hispanic or Latino	9 (100)	1 (100)	10 (100)

HPP = hypophosphatasia; 6MWT = 6‐Minute Walk Test; BL = baseline; SD = one‐third standard deviation method (SD × 1/3); NA = not applicable; SEM = standard error of measurement method; MCID = minimal clinically important difference.

^a^ One child was generally non‐ambulatory and was unable to walk the full 6 min at BL; this patient was excluded from the SD and SEM MCID analyses and the test‐retest validity analysis. Another child completed the 6MWT at BL but did not have data recorded (reason not specified); this child was not included in the calculation of the r value used for the SEM MCID analysis or in the test‐retest reliability analysis.

^b^ Two adolescents were unable to complete the 6MWT at both screening and BL. One adolescent could not walk at a self‐selected speed (parents pulled him along) and could not understand test instructions because of cognitive impairment. The other adolescent could not ambulate functionally without his walker, which he did not have with him at the screening visit and was unable to walk the full 6 min with the walker at the BL visit. These patients were excluded from the SD and SEM MCID analyses and test‐retest reliability analysis.

^c^ One adult was unable to complete the 6MWT at both screening and BL because he was generally non‐ambulatory and used a wheelchair for mobility. This patient was excluded from the SD and SEM MCID analyses and the test‐retest reliability analysis.

The median (minimum, maximum) distance walked was 355 (190, 491) m for children completing the 6MWT at either screening or baseline (*n* = 12). For children completing the 6MWT at both time points (*n* = 11), median (minimum, maximum) distance walked was 380 (188, 557) m at screening and 350 (190, 491) m at baseline. The median (minimum, maximum) percent predicted distance walked compared with healthy sex‐, age‐, and height‐matched children was 64.3% (29%, 87%) at screening and 61.0% (29%, 82%) at baseline.

**Figure 1:**
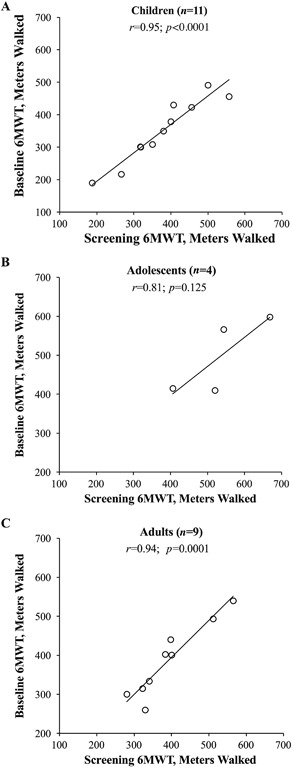
Test‐retest reliability: Pearson correlations (*r*) between distances walked on the 6MWT at screening and baseline in (*A*) children, (*B*) adolescents, and (*C*) adults with HPP. 6MWT = 6‐Minute Walk Test; HPP = hypophosphatasia.

The test‐retest reliability of the 6MWT was high; the Pearson's correlation coefficient (*r*) between distance walked at screening versus distance at baseline was 0.95 (*p* < 0.0001) (Fig. 1 *A*; Table 3). The MCID for the 6MWT in children with HPP was estimated at 20.6 m using the SEM method and 30.8 m using the SD method (Table 3).

**Table 3:** 6MWT Test‐Retest Reliability, Distance Walked, and MCID for Patients With HPP by Age Group

	Children (age 5–12 years at enrollment)	Adolescents (age 13–17 years at enrollment)	Adults (age ≥18 years at enrollment)
**Test‐retest reliability** ^a^	***n* = 11**	***n* = 4**	***n* = 9**
Distance walked (m), mean ± SD			
Screening	376.5 ± 105.3	534.5 ± 107.5	391.9 ± 92.5
BL	349.5 ± 96.8	497.3 ± 98.8	387.2 ± 93.3
*r* ^b^	0.95	0.81	0.94
*p* value	<0.0001	0.125	0.0001
**Composite BL distance walked (m)** ^c^	***n* = 12**	***n* = 4**	***n* = 9**
Mean ± SD	350.4 ± 92.3	497.3 ± 98.8	387.2 ± 93.3
**MCID (m)**	*n* = 12	*n* = 4	*n* = 9
SEM method	20.6	43.0	22.8
SD method	30.8	32.9	31.1

6MWT = 6‐Minute Walk Test; MCID = minimal clinically important difference; HPP = hypophosphatasia; SD = standard deviation; BL = baseline; SEM = standard error of measurement.

^a^ Only patients who completed the 6MWT at BL and screening were included in the correlation analysis.

^b^ Pearson correlation between 6MWT distance‐walked at screening versus BL visits.

^c^ Screening values were used when BL values were missing.

**Figure 2:**
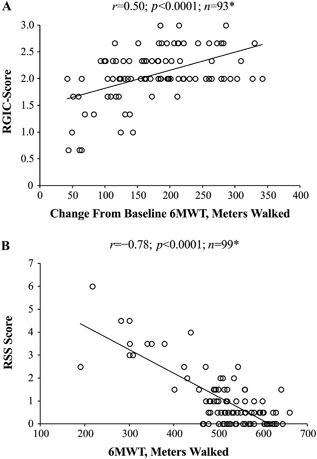
Pearson correlations (*r*) between the 6MWT and measures of skeletal disease in children with HPP: (*A*) RGI‐C score versus change from baseline in 6MWT distance walked and (*B*) RSS score versus 6MWT distance walked. *n represents repeated observations of the same patient pool over time, not the number of patients. 6MWT = 6‐Minute Walk Test; HPP = hypophosphatasia; RGI‐C = Radiographic Global Impression of Change; RSS = Rickets Severity Scale.

Distance walked during the 6MWT correlated with scores on measures of skeletal disease (RGI‐C and RSS). There was a positive linear relationship between the change from baseline in 6MWT distance walked and improvement in RGI‐C score (*r* = 0.50; *p* < 0.0001; Fig. 2 *A*) and a negative linear relationship between distance walked and RSS score (*r* =–0.78; *p* < 0.0001; Fig. 2 *B*), such that distance walked increased as the severity of skeletal disease decreased.

**Figure 3:**
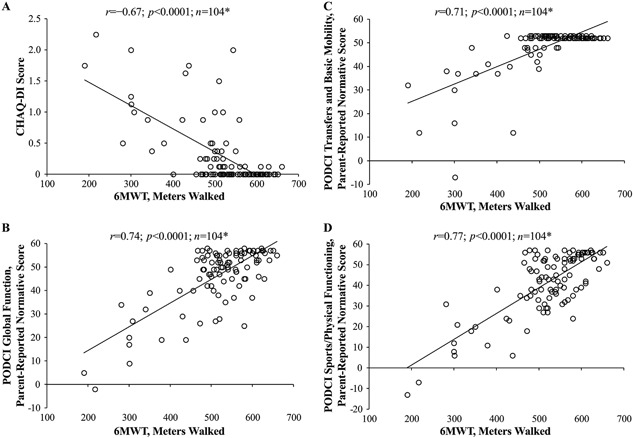
Pearson correlations (*r*) between 6MWT distance walked and the following parent‐reported measures of disability and functional status in children with HPP: (*A*) CHAQ‐DI score; (*B*) PODCI Global Function score; (*C*) PODCI Transfers and Basic Mobility score; and (*D*) PODCI Sports/Physical Functioning score. *n represents repeated observations of the same patient pool over time, not the number of patients. 6MWT = 6‐Minute Walk Test; CHAQ‐DI = Childhood Health Assessment Questionnaire Disability Index; HPP = hypophosphatasia; PODCI = Pediatric Outcomes Data Collection Instrument.

**Figure 4:**
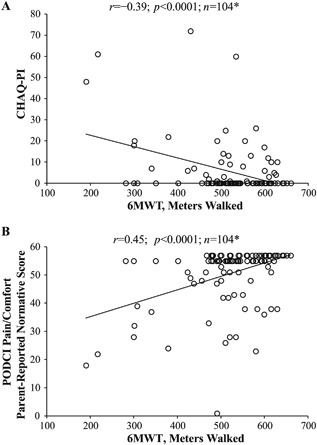
Pearson correlations (*r*) between 6MWT distance walked and the following parent‐reported measures of pain in children with HPP: (*A*) CHAQ‐PI score and (*B*) PODCI Pain/Comfort score. *n represents repeated observations of the same patient pool over time, not the number of patients. 6MWT = 6‐Minute Walk Test; CHAQ‐PI = Childhood Health Assessment Questionnaire Pain Index; HPP = hypophosphatasia; PODCI = Pediatric Outcomes Data Collection Instrument.

The 6MWT distance walked also correlated with scores on parent‐reported measures of disability (CHAQ‐DI) and ability to function in activities of daily living (PODCI subscales). A negative linear relationship was found between distance walked and CHAQ‐DI score (*r* = −0.67; *p* < 0.0001; Fig. 3 *A*). Positive linear relationships were found between distance walked and parent‐reported normative scores on each of the following PODCI subscales: Global Function (*r* = 0.74; *p* < 0.0001; Fig. 3 *B*), Transfers and Basic Mobility (*r* = 0.71; *p* < 0.0001; Fig. 3 *C*), and Sports and Physical Functioning (*r* = 0.77; *p* < 0.0001; Fig. 3 *D*). The 6MWT also correlated with parent‐reported measures of pain. Distance walked had a negative correlation with the CHAQ‐PI (*r* =–0.39; *p* < 0.0001; Fig. 4 *A*) and a positive correlation with the PODCI Pain/Comfort subscale (*r* = 0.45; *p* < 0.0001; Fig. 4 *B*).

#### Adolescents

Disposition and demographics of the six adolescents with HPP enrolled in study 2 are summarized in Table 2. Two adolescent patients used assistive devices (orthotic shoe inserts, *n* = 1; bilateral ankle‐foot orthoses, *n* = 1) during the 6MWT at screening and baseline.

The median (minimum, maximum) distance walked by adolescents completing the 6MWT at both time points was 532 (406, 668) m at screening and 491 (410, 598) m at baseline. The median (minimum, maximum) percent predicted distance walked was 78.3% (61%, 95%) at screening and 73.7% (59%, 85%) at baseline.

The test‐retest correlation between the distance walked at screening vs. baseline was positive (*r* = 0.81) but not statistically significant in adolescents (*p* = 0.125; Fig. 1 *B*; Table 3). The estimated MCID for the 6MWT in adolescents with HPP was 43.0 m using the SEM method and 32.9 m using the SD method (Table 3).

**Figure 5:**
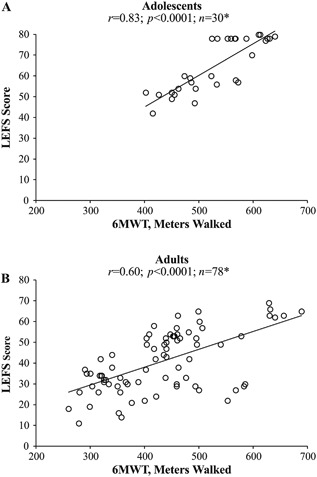
Pearson correlations (*r*) between 6MWT distance walked and LEFS scores in (*A*) adolescents (age 13 to 17 years at enrollment) and (*B*) adults (age ≥18 years at enrollment) with HPP. *n represents repeated observations of the same patient pool over time, not the number of patients. 6MWT = 6‐Minute Walk Test; HPP = hypophosphatasia; LEFS = Lower Extremity Function Scale.

**Figure 6:**
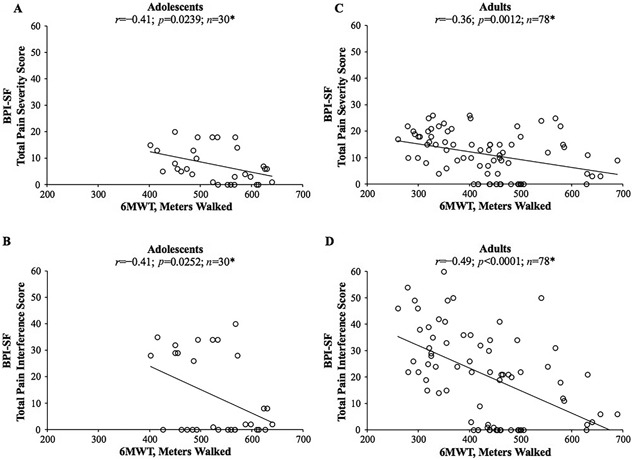
Pearson correlations (*r*) between 6MWT distance walked and (*A*) BPI‐SF total pain severity scores in adolescents (age 13 to 17 years at enrollment); (*B*) BPI‐SF total pain interference scores in adolescents; (*C*) BPI‐SF total pain severity scores in adults (age ≥18 years at enrollment); and (*D*) BPI‐SF total pain interference scores in adults with HPP. *n represents repeated observations of the same patient pool over time, not the number of patients. 6MWT = 6‐Minute Walk Test; HPP = hypophosphatasia; BPI‐SF = Brief Pain Inventory‐Short Form.

Distance walked during the 6MWT had a positive linear relationship with LEFS score (*r* = 0.83; *p* < 0.0001; Fig. 5 *A*) and negative linear relationships with the BPI‐SF total pain severity score (*r* = −0.41; *p* = 0.0239; Fig. 6 *A*) and total pain interference score (*r* = −0.41; *p* = 0.0252; Fig. 6 *B*).

#### Adults

Disposition and demographics of the 10 adults with HPP enrolled in study 2 are summarized in Table 2. Assistive devices were used by two adults at screening (orthotics in shoes and wheeled walker, *n* = 1; orthotics in shoes, *n* = 1) and three adults at baseline (orthotics in shoes and wheeled walker, *n* = 1; orthotics in shoes, *n* = 1; boot on left foot, *n* = 1).

The median (minimum, maximum) distance walked by adults completing the 6MWT at both time points was 383 (280, 565) m at screening and 401 (260, 540) m at baseline. The median (minimum, maximum) percent predicted distance walked was 73.4% (54%, 106%) at screening and 77.3% (52%, 101%) at baseline.

The test‐retest reliability was high in adults (*r* = 0.94; *p* = 0.0001; Fig. 1 *C*; Table 3). The MCID was estimated at 22.8 m using the SEM method and 31.1 m using the SD method (Table 3).

The 6MWT distance walked had a positive linear relationship with LEFS score (*r* = 0.60; *p* < 0.0001; Fig. 5 *B*), a negative linear relationship with BPI‐SF total pain severity score (*r* = −0.36; *p* = 0.0012; Fig. 6 *C*), and a negative linear relationship with BPI‐SF total pain interference score (*r* = −0.49; *p* < 0.0001; Fig. 6 *D*).

## Discussion

This analysis of data from two clinical studies of asfotase alfa is the first to establish the reliability, MCIDs, and validity of the 6MWT in patients with HPP. Test‐retest reliability of the 6MWT was high (*r* > 0.8) in children, adolescents, and adults with HPP. The most conservative estimates of the MCID were 31 m for children, 43 m for adolescents, and 31 m for adults. These values are consistent with 6MWT MCIDs estimated for other patient populations. In children and adolescents, MCID estimates range from 26 to 32 m in males with Duchenne muscular dystrophy (age 4 to 12 years: 26 m^40^; age 5 to 20 years: 29 to 32 m^14^) and from 17 to 23 m in ambulatory patients with cerebral palsy (age 4 to 18 years).^41^ Estimates of the MCID in adults with pulmonary and cardiovascular diseases range from 22 to 37 m (chronic obstructive pulmonary disease: 25 to 35 m^42^, ^43^, ^44^; coronary artery disease: 25 m^45^; idiopathic pulmonary fibrosis: 22 to 37 m^46^; non–cystic fibrosis bronchiectasis: 25 m^47^; and diffuse parenchymal lung disease: 30 to 33 m^48^). The MCID in this HPP population was lower among children and adults than adolescents. This would be expected, as normative values for the average 6MWT distance walked increase with height and age in healthy children, peak in adolescents and young adults, and decrease with increasing age in older adults.^29^, ^30^

The median 6MWT distances walked at screening and baseline by children (380 and 350 m) and adolescents (532 and 491 m) with HPP who completed the 6MWT were lower than distances reported for healthy children and adolescents (mean: 618 m)^49^ and for those with conditions such as cystic fibrosis (mean: 556 to 640 m)^50^ and obesity (mean: 571 m),^51^ reflecting the substantial burden of HPP on mobility. Similarly, the median 6MWT distances walked by adults with HPP (screening: 383 m; baseline: 401 m) were lower than values reported for healthy adults (mean: 614 m).^52^ HPP is associated with bone deformities and nontraumatic slowly healing fractures, which can affect a patient's ability to ambulate.^4^, ^6^, ^7^, ^53^, ^54^, ^55^, ^56^ Muscular complications of HPP (eg, muscle pain and weakness) may further impair endurance and walking speed. It is not clear how low TNSALP activity leads to muscular complications in HPP.^4^ Studies in a murine model of HPP suggest that elevated inorganic pyrophosphate levels may cause muscle weakness.^57^ The bone deformity, fractures, and muscular weakness in HPP are clinically similar to those seen in patients with osteogenesis imperfecta (OI). One study used the 1‐min walk test to evaluate the effectiveness of a physiotherapy strengthening program on mobility in children with OI.^58^ It should also be considered that when using the 6MWT to evaluate the effectiveness of treatments for musculoskeletal disorders such as HPP, improved mobility may be a result of improvements of systemic manifestations of the disease in addition to bone deformities, such as pain.

Correlation analyses showed the concurrent validity of the 6MWT with clinically relevant measures of skeletal disease and parent‐reported function, disability, and pain in children with HPP, and patient‐reported lower extremity function and pain in adolescents and adults with HPP. These findings are not unexpected, as musculoskeletal complications in HPP can affect a patient's ability to perform activities of daily living. For example, children may be unable to successfully navigate the school environment or participate in physical education and recreational activities and adults may be unable to work. Participation in these activities may also be affected by kinesiophobia and other psychosocial factors related to chronic pain.^59^, ^60^, ^61^ Distance walked on the 6MWT has also been shown to correlate with functional status and quality‐of‐life measures in patients with cardiac and pulmonary diseases.^62^, ^63^, ^64^

This investigation was performed as a post hoc analysis, which has some limitations, as these analyses were not prospectively defined. The sample sizes for these analyses were small, with the number of patients in each age cohort limited to 11 children, four adolescents, and nine adults. In particular, the low number of adolescent patients limited the statistical power to detect a significant correlation in the test‐retest analysis in this age group. As such, the data for the adolescents group should be interpreted with caution, and future analyses of this age group are warranted. In addition, the influence of assistive devices, which are permitted during the 6MWT,^(8)^ was not assessed in this analysis.

## Conclusions

The 6MWT is a reliable and valid measure in children, adolescents, and adults with HPP signs and symptoms first occurring before 18 years of age. The conservative estimate of the MCID for 6MWT distance walked is 31 m for children, 43 m for adolescents, and 31 m for adults with HPP. The 6MWT showed concurrent validity with clinically relevant measures of skeletal disease and parent‐reported function and disability in children, and patient‐reported function and pain in adolescents and adults with HPP. This well‐accepted measure of physical activity can be used to assess meaningful changes in mobility occurring as a consequence of disease progression or with treatment in patients with HPP.

## Disclosures

DP was a consultant for Alexion Pharmaceuticals, Inc., and at the time of the study had received funding and travel support from Alexion for consulting and participating on advisory boards. ICT and SM are employees of and may own stock/options in Alexion Pharmaceuticals, Inc., which sponsored the study. GL and HG were employees of and may own stock/options in Alexion Pharmaceuticals, Inc. SLL is a consultant for Alexion Pharmaceuticals, Inc., and has received funding and travel support from Alexion for consulting and participating on advisory boards.
